# Characterization of Salivary and Plasma Metabolites as Biomarkers for HCC: A Pilot Study

**DOI:** 10.3390/cancers15184527

**Published:** 2023-09-12

**Authors:** Courtney E. Hershberger, Roma Raj, Arshiya Mariam, Nihal Aykun, Daniela S. Allende, Mark Brown, Federico Aucejo, Daniel M. Rotroff

**Affiliations:** 1 Department of Quantitative Health Sciences, Lerner Research Institute, Cleveland Clinic, Cleveland, OH 44195, USA; 2 Center for Quantitative Metabolic Research, Cleveland Clinic, Cleveland, OH 44195, USA; 3 Digestive Disease and Surgery Institute, Cleveland Clinic, Cleveland, OH 44195, USA; 4 Department of Cancer Biology, Lerner Research Institute, Cleveland Clinic, Cleveland, OH 44195, USA; 5 Center for Microbiome and Human Health, Cleveland Clinic, Cleveland, OH 44195, USA; 6 Endocrinology and Metabolism Institute, Cleveland Clinic, Cleveland, OH 44195, USA

**Keywords:** metabolomics, biomarkers, cancer, hepatocellular carcinoma, liver disease

## Abstract

**Simple Summary:**

Hepatocellular carcinoma is diagnosed using ultrasound and blood-biomarker AFP, which can miss up to 40% of diagnoses, delaying the treatment of these patients. The aim of our study was to identify metabolites that are associated with HCC in blood and saliva, and compare the metabolic profiles of these two biofluids. Malonic acid was the only metabolite that was correlated in paired blood and saliva samples, and it was also found to be associated with HCC in saliva. In total, 6 metabolites in saliva and 10 different metabolites in plasma were found to be associated with HCC. The ‘Citric Acid Cycle’ pathway was deregulated in both the blood and saliva of patients with HCC. This suggests that plasma and saliva offer unique sources of metabolite biomarkers, but these metabolites converge on a common pathway in cell metabolism.

**Abstract:**

(1) Background: The incidence of hepatocellular carcinoma (HCC) is rising, and current screening methods lack sensitivity. This study aimed to identify distinct and overlapping metabolites in saliva and plasma that are significantly associated with HCC. (2) Methods: Saliva samples were collected from 42 individuals (HCC = 16, cirrhosis = 12, healthy = 14), with plasma samples from 22 (HCC = 14, cirrhosis = 2, healthy = 6). We performed untargeted mass spectrometry on blood and plasma, tested metabolites for associations with HCC or cirrhosis using a logistic regression, and identified enriched pathways with Metaboanalyst. Pearson’s correlation was employed to test for correlations between salivary and plasma metabolites. (3) Results: Six salivary metabolites (1-hexadecanol, isooctanol, malonic acid, N-acetyl-valine, octadecanol, and succinic acid) and ten plasma metabolites (glycine, 3-(4-hydroxyphenyl)propionic acid, aconitic acid, isocitric acid, tagatose, cellobiose, fucose, glyceric acid, isocitric acid, isothreonic acid, and phenylacetic acid) were associated with HCC. Malonic acid was correlated between the paired saliva and plasma samples. Pathway analysis highlighted deregulation of the ‘The Citric Acid Cycle’ in both biospecimens. (4) Conclusions: Our study suggests that salivary and plasma metabolites may serve as independent sources for HCC detection. Despite the lack of correlation between individual metabolites, they converge on ‘The Citric Acid Cycle’ pathway, implicated in HCC pathogenesis.

## 1. Introduction

Liver cancer is one of the leading causes of cancer-related mortality worldwide. Its incidence is estimated to rise to >1 million cases by 2025 [1]. Hepatocellular carcinoma (HCC) remains the most common type of primary liver cancer, accounting for more than 90% of the cases [1]. Chronic infection with hepatitis B and C viruses dominates the etiology of HCC, followed by alcoholic cirrhosis [1]. More recently, there has been an emergence of HCC in the setting of non-alcoholic steatohepatitis (NASH) and non-alcoholic fatty liver disease (NAFLD), which are associated with diabetes or metabolic syndrome.

The surgical removal of tumors, either by resection or liver transplantation, offers the best long-term outcomes and is the treatment of choice in early-stage disease [2]. However, most cases are diagnosed at an advanced stage, where surgical resection does not impact outcome and is contraindicated. These patients undergo systemic or liver-directed chemo- or radiotherapeutic treatments and have a prognosis of <1 year survival [2], emphasizing the need for early detection of HCC. 

Current surveillance strategies in patients with cirrhosis involve ultrasound every 6 months, with or without monitoring alpha-fetoprotein (AFP) levels, the existing clinical biomarker of disease activity for HCC [3]. The detection of HCC with AFP alone has a sensitivity of 61% and specificity of 86%, and adding ultrasound marginally increases the sensitivity and specificity to 62% and 88%, respectively [3]. This lack of sensitivity means that many patients with HCC go undetected. Early detection has been shown to improve HCC outcomes [4], and there is a dire need for more accurate tools to screen and diagnose HCC so that timely treatment can be initiated. More sensitive biomarkers that could be used for surveillance in these patients would help to decrease the number of missed diagnoses, improve early detection of the disease, and aid the successful employment of curative treatment options before progression to advanced stages. 

Metabolites in saliva have shown promise as non-invasive biomarkers of breast, prostate, and oral cancers [5,6,7]. We previously investigated the potential use of salivary biomarkers to detect HCC in a cohort of 110 individuals with HCC, cirrhosis, or healthy livers. We generated a predictive model using four biomarker metabolites with 85% sensitivity and 92% specificity [8].

Here, we extend this work to perform untargeted metabolomics on paired plasma (*N* = 22) and saliva (*N* = 42) samples from individuals with HCC, cirrhosis, and healthy livers. We sought to characterize the role of metabolites in HCC through a comparative study of salivary and plasma metabolic profiles, which demonstrated distinct profiles from one another, indicating that metabolic signatures in saliva and blood may provide improved detection over either biospecimen alone.

## 2. Materials and Methods

### 2.1. Subject Recruitment 

Saliva and blood samples were collected from a clinical cohort of 42 adult patients seen at the Cleveland Clinic (Cleveland, OH, USA) between 2018 and 2021 with cirrhosis (*N* = 12, 2) or HCC (*N* = 16, 14) that underwent liver transplantation for HCC and/or cirrhosis, surgical resection for HCC, or liver biopsy with confirmed cirrhosis and/or HCC. In addition to an initial assessment with clinical presentation and imaging, a histopathological assessment was performed to confirm the diagnoses of HCC and cirrhosis as part of the standard of care. Patients attending treatment for hernia with no history of liver disease or liver cancer were recruited as healthy subjects (*N* = 14, *N* = 6). The clinical characteristics of the study participants can be found in Table 1. All participants provided informed written consent, the study conformed to the ethical guidelines of the 1975 Declaration of Helsinki, and it was approved by the Cleveland Clinic IRB (IRB #10-347).

### 2.2. Saliva Collection and Gas Chromatography Mass Spectrometry 

During a scheduled visit with their physician, a saliva sample was collected from each subject after a standard mouth rinse, using the DNA Genotek OMNIgene ORAL OM-610 (Ottawa, ON, Canada) saliva collection kit. The samples were then transferred to cryovials for storage at −80 °C. Blood samples were also collected from 22 of the 42 subjects. Blood samples were collected in EDTA tubes and stored at room temperature for no more than 60 min and plasma was extracted by centrifugation of the blood samples at 3000 rpm at 4 °C for 20 min. The plasma samples were washed with 1.4 mL Hanks’ Balanced Salt Solution and stored in a cryovial tubes at −80 °C. The saliva and plasma samples were subjected to untargeted gas chromatography time of flight mass spectrometry (GC-TOF MS) at the West Coast Metabolomics Center (Davis, CA, USA) as described previously [8]: A Leco Pegasus IV mass spectrometer was used with unit mass resolution at 17 spectra s^−1^ from 80–500 Da at −70 eV ionization energy and 1800 V detector voltage with a 230 °C transfer line and a 250 °C ion source. The relative abundance of each metabolite was calculated using ChromaTOF version 2.32 and the BinBase algorithm (rtx5) (see Supplementary Methods).

### 2.3. Data Processing and Quality Control 

The metabolite abundances were reported for 246 metabolites in saliva and plasma. Metabolites with <10% high-quality spectra were excluded, leaving 202 salivary metabolites and 156 plasma metabolites, with 112 metabolites being represented in both biofluids (see Supplementary Methods). Because missing metabolite values are often below the limit of detection, a metabolite lacking a detected peak was imputed using the quantification mass at the target retention index after subtracting the local minimum noise. Metabolites that did not have high-quality spectra counts in at least 10% of their respective biofluid were not assessed for associations with disease states (Supplementary Table S1).

The differences in the number of reported identified metabolites are likely due to the sample sizes of each group (saliva = 42, plasma = 22). The relative abundances of the metabolites were right-skewed and were therefore log-transformed and scaled and centered across the cohort. The transformation resulted in normal distributions of the relative abundances of the metabolites (Supplementary Figures S1 and S2). To ensure that age was not a confounding factor, we performed linear regression to test for associations between age and each metabolite while adjusting for disease status. We then adjusted for multiple hypothesis testing using an FDR approach.

### 2.4. Metabolite Associations with HCC

We used logistic regression to test for statistically significant associations between the relative abundance of each metabolite and HCC diagnosis. We performed pairwise comparisons for each salivary and plasma metabolite across our three cohorts; HCC vs. healthy, HCC vs. cirrhosis, and HCC vs. non-HCC (cirrhosis cohort + healthy cohort). The *p* values were adjusted for multiple hypothesis testing using a false discovery rate (FDR) [9] approach, and an FDR *p* < 0.10 was used as the threshold for statistical significance.

### 2.5. Correlation of Metabolite Relative Abundance between Plasma and Saliva Samples 

To characterize the overlap of metabolic landscapes between the two types of biospecimens, the relative abundances of each metabolite that were reported in both saliva and plasma were compared using Pearson’s correlation. All *p* values were adjusted for multiple hypothesis testing using the FDR approach [9], and an FDR *p* < 0.10 was used as the threshold for statistical significance.

### 2.6. Pathway Analysis 

To identify biological pathways that may be impacted by metabolites that displayed differential abundance between disease cohorts, metabolites with a nominal *p* < 0.05 for the pairwise comparisons in saliva and/or plasma were each tested for pathway association using the MetaboAnalyst webtool, which employs Metabolite Set Enrichment Analysis (MSEA) to perform over representation analysis (ORA) [10]. MSEA contains a library of ~1600 predefined metabolite sets covering metabolite pathways and disease states derived from the literature, PubMed, Human Metabolome Database (HMDB), Metabolic Information Center (MIC), and Small Molecular Pathway Database (SMPDB). We performed ORA to test whether the nominally significant metabolites were found within a metabolite set more often than expected by chance. The input was a list of significant metabolites (*p* < 0.05), and the *p* value indicates the probability of identifying that number of metabolites in the metabolite set by chance, using a hypergeometric distribution (see Supplementary Methods). After adjustment for multiple hypothesis testing, FDR *p* < 0.10 was used as the threshold for statistical significance [9]. 

## 3. Results

### 3.1. Salivary Metabolite Associations with HCC 

**Figure 1:**
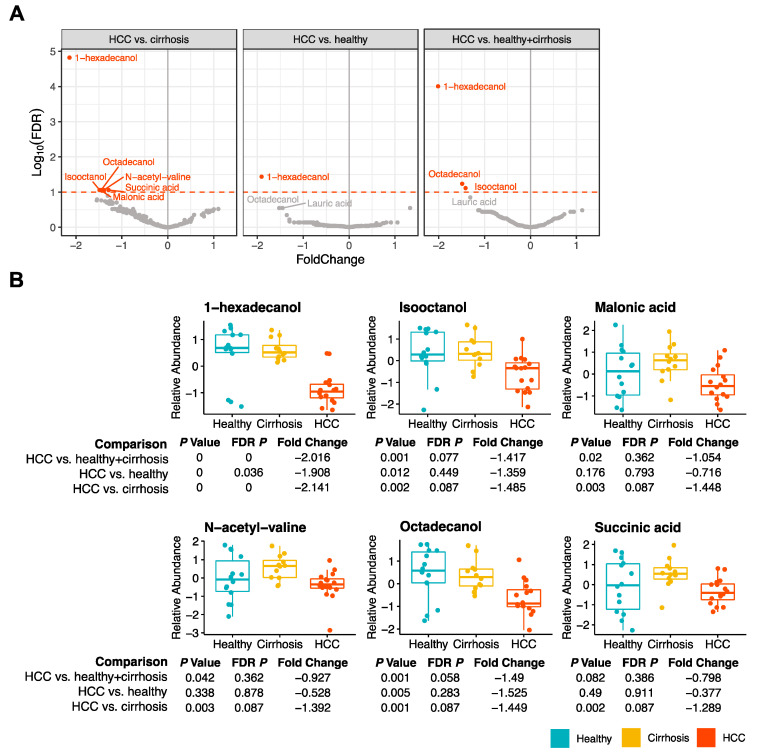
Untargeted metabolomics of saliva samples from individuals with HCC, cirrhosis, and healthy livers. (**A**) Volcano plots depicting the fold change and false discovery rate for metabolites associated with HCC compared to cirrhosis, HCC compared to healthy livers, and HCC compared to non-HCC (cirrhosis and healthy livers). Metabolites significantly associated with each outcome are highlighted in red and FDR *p* of 0.1 is marked with a red dotted line. (**B**) Boxplots depicting the relative abundance of each metabolite significantly associated with HCC (FDR *p* < 0.1) for all pairwise comparisons.

A total of 202 metabolites were quantified in each of the 42 saliva samples and assessed for associations with HCC (Supplementary Table S2). Two metabolites, 1-hexadecanol (FDR *p* < 0.001) and isooctanol (FDR *p* = 0.077), were significantly decreased in the saliva of those with HCC when compared to healthy + cirrhosis (FDR *p* < 0.1) (Figure 1A,B). Five metabolites, including 1-hexadecanol (FDR *p* < 0.001), isooctanol (FDR *p* = 0.087), malonic acid (FDR *p* = 0.087), N-acetyl-valine (FDR *p* = 0.087), and succinic acid (FDR *p* = 0.087), were significantly decreased in the saliva of those with HCC compared to healthy + cirrhosis (FDR *p* < 0.1) (Figure 1A,B). We found that 1-hexadecanol was significantly decreased in the saliva of individuals with HCC when compared to individuals with cirrhosis (FDR *p* = 0.036) (Figure 1A,B). There were no salivary metabolites found to be associated with age.

### 3.2. Plasma Metabolite Associations with HCC 

**Figure 2:**
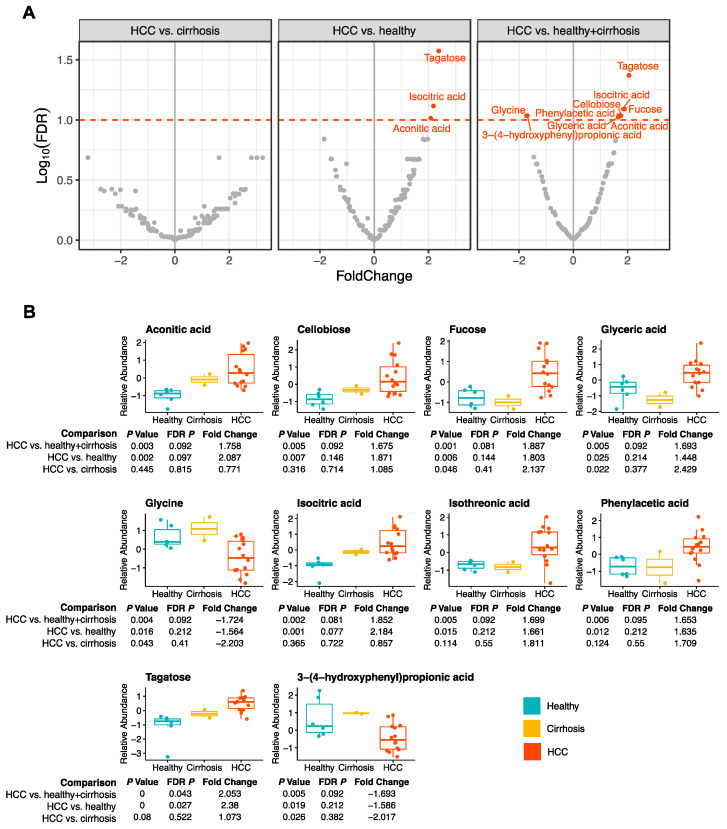
Untargeted metabolomics of plasma samples from individuals with HCC, cirrhosis, and healthy livers. (**A**) Volcano plots depicting the fold change and false discovery rate for metabolites associated with HCC compared to cirrhosis, HCC compared to healthy livers, and HCC compared to non-HCC (cirrhosis and healthy livers). Ten metabolites with an FDR *p* < 0.1 (red dotted line) are highlighted. (**B**) Ten boxplots depicting the relative abundance of the metabolites that are significantly associated with HCC in at least one pairwise comparison.

The paired plasma samples collected from 22 study participants were also assessed via untargeted mass spectrometry, yielding relative abundances for 156 metabolites. Ten metabolites were significantly associated with HCC in plasma in at least one comparison (Figure 2A) (FDR *p* < 0.10) (Supplementary Table S3).

Glycine (FDR *p* = 0.092) and 3-(4-hydroxyphenyl)propionic acid (FDR *p =* 0.092) were significantly decreased in HCC when compared to plasma from healthy + cirrhosis. Three metabolites, aconitic acid (FDR *p =* 0.097), isocitric acid (FDR *p =* 0.077), and tagatose (FDR *p* = 0.27) were significantly higher in plasma from those with HCC when compared to healthy patients (Figure 2). Eight metabolites, including aconitic acid (FDR *p* = 0.092), cellobiose (FDR *p* = 0.092), fucose (FDR *p* = 0.081), glyceric acid (FDR *p* = 0.092), isocitric acid (FDR *p* = 0.081), isothreonic acid (FDR *p* = 0.092), phenylacetic acid (FDR *p* = 0.095), and tagatose (FDR *p* = 0.043), were significantly higher in in plasma from those with HCC when compared to healthy + cirrhosis (Figure 2). We found that 153/156 plasma metabolites were not associated with age. The three metabolites that were significantly associated with age in plasma were 3,6-1nhydro-D-galactose, cysteine, and beta-alanine. These metabolites were not significantly associated with disease state.

### 3.3. Correlation of Relative Abundances between Plasma and Salivary Metabolites

**Figure 3:**
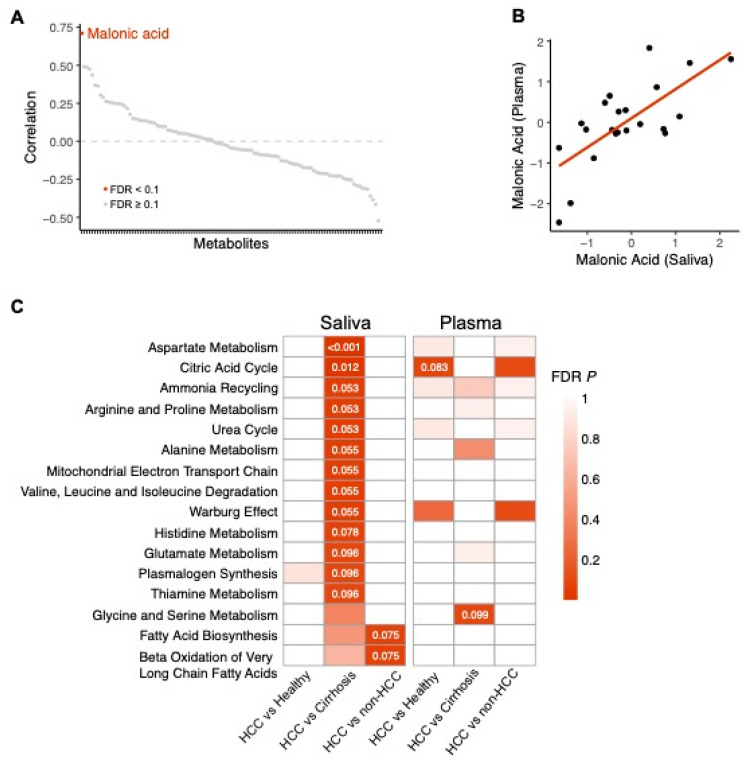
Comparison of metabolites between saliva and plasma samples. (**A**) Scatterplot depicting the correlation between each metabolite in saliva and plasma samples. Malonic acid, the only metabolite with an FDR *p* < 0.1, is highlighted in red. (**B**) The relative abundance of malonic acid is significantly correlated between saliva and plasma samples. (**C**) Heatmap depicting FDR *p* values for pathway analysis performed on metabolites associated with HCC (*p* < 0.05) in saliva and plasma samples. FDR *p* values are displayed for significant pathways. (FDR *p* > 0.1).

Out of the 121 metabolites that were quantified in both the saliva and the plasma samples, the relative abundance of malonic acid was the only metabolite that was significantly correlated between the two biospecimens (*FDR p =* 0.02, R = 0.71) (Figure 3A,B) (Supplementary Table S4).

### 3.4. Pathway Analysis

Forty-three salivary metabolites and forty-five plasma metabolites were associated with HCC in at least one of the comparisons, with a nominal *p* value < 0.05. These plasma metabolites were enriched in two metabolic pathways, and the salivary metabolites were found to be enriched in 15 metabolic pathways (Figure 3C).

In saliva, the 29 metabolites that were associated with HCC compared to cirrhosis were enriched for ‘aspartate metabolism’ (FDR *p* < 0.001), ‘citric acid cycle’ (FDR *p* = 0.012), ‘ammonia recycling’ (FDR *p* = 0.053), ‘arginine and proline metabolism’ (FDR *p* = 0.053), ‘urea cycle’ (FDR *p* = 0.053), ‘alanine metabolism’ (FDR *p* = 0.055), ‘mitochondrial electron transport chain’ (FDR *p* = 0.055), ‘Warburg effect’ (FDR *p* = 0.055), ‘histidine metabolism’ (FDR *p* = 0.078), ‘glutamate metabolism’ (FDR *p* = 0.096), ‘plasmalogen synthesis’ (FDR *p* = 0.096), and ‘thiamine metabolism’ (FDR *p* = 0.096) (Figure 3C).

In saliva, the 25 metabolites that were associated with HCC compared to healthy + cirrhosis were enriched for ‘fatty acid biosynthesis (FDR *p* = 0.075) and ‘beta oxidation of very long chain fatty acids’ (FDR *p* = 0.075) (Figure 3C).

In plasma, 25 metabolites associated with HCC when compared to healthy individuals were enriched for the ‘citric acid cycle’ (FDR *p* = 0.083) (Figure 3C). Finally, the 28 metabolites associated with HCC when compared to both cirrhosis and healthy were enriched ‘glycine and serine metabolism’ (FDR *p =* 0.099) (Figure 3C). 

## 4. Discussion

Due to the rising incidence of HCC, there is a dire need for an easily accessible, inexpensive, and noninvasive method of screening patients at risk of developing HCC with improved sensitivity. We previously generated a salivary metabolite signature that can distinguish HCC from individuals with healthy or cirrhotic livers. Although salivary metabolites are emerging as a potentially valuable biospecimen for biomarkers of HCC, it was unclear whether the saliva presented a snapshot of the metabolic profile in plasma, or if they each presented independent sources of information. In this study, we characterized the similarities and differences between the metabolite profiles of paired saliva and plasma samples in patients with and without liver disease, including HCC.

### 4.1. Metabolites in Saliva Associated with HCC

1-hexadecanol, the most significant metabolite detected in saliva, is a long-chain fatty acid that had been previously identified as an exhaled VOC that can differentiate cirrhosis from chronic liver disease [11]. In breath, 1-hexadecanol is lower in individuals with cirrhosis when compared to chronic liver disease [11], and in this study, it was found to be significantly lower in individuals with HCC when compared to both those with cirrhotic and healthy livers. 

Malonic acid is a dicarboxylic acid that participates in fatty acid biosynthesis and aspartate metabolism, and although it has been investigated in the context of colon cancer, it is not known to be associated with HCC [12,13]. Similarly, N-acetyl-valine, an N-acyl-alpha amino acid, has been detected in fecal samples for colon cancer [12,13], but this is the first time that these two metabolites have been shown to be associated with HCC.

Succinic acid is a dicarboxylic acid that is a component of the TCA cycle. Salivary levels of succinic acid have been combined with other metabolites to create a model that has been shown to be able to distinguish hepatitis B from healthy controls [14]. Succinic acid in the plasma has shown discriminatory capacity for endstage liver disease and HCC [15]. In vitro, succinic acid was associated with resistance to sorafenib, a treatment for advanced-stage HCC [16].

### 4.2. Metabolites in Plasma Associated with HCC

We quantified the plasma metabolic profiles of 22 individuals in our cohort and identified ten plasma metabolites that were significantly associated with HCC when compared to healthy and/or cirrhosis. Importantly, there was no significant overlap between these ten metabolites and the six that were associated with HCC in saliva, suggesting that plasma and saliva may present unique information regarding the presence of HCC.

In plasma, the abundance of glycine was significantly lower in HCC compared to healthy + cirrhosis. Glycine is an amino acid synthesized in the liver that has been detected in multiple HCC-related metabolomics studies of plasma and serum [17]. Concordantly, in a cohort of 128 subjects (63 HCC and 65 cirrhosis) [18], decreased levels of glycine were observed in those with HCC. Furthermore, glycine was one of 11 metabolites selected in a predictive model for discriminating HCC from cirrhosis (AUC = 0.81) [18]. However, the plasma and serum levels of glycine in studies comparing HVC-HCC and HCV-cirrhosis have shown increased glycine in the HCV-HCC group [19,20]. Among individuals who are diagnosed with HCC, circulating glycine levels have been shown to be correlated with HCC progression, with glycine levels decreased in the serum of individuals with early HCC compared to advanced HCC [21].

We found levels of isocitric acid to be elevated in the plasma of individuals with HCC. Interestingly, high levels of isocitric acid, glutamic acid, and aspartic acid in plasma can differentiate individuals with NASH from those with NAFLD [22]. This suggests a potential continuum along which plasma levels of isocitric acid correlate with liver disease severity. This model also included aconitic acid, a metabolite found to be high in our HCC cohort. Furthermore, aconitic acid levels in urine have been shown to be predictive of HCC recurrence after surgical resection [23].

Phenylacetic acid is a component of a proposed HCC therapy, AS2-1, that initially showed promise for delaying HCC recurrence by decreasing cell proliferation [24].

Tagatose was elevated in the plasma of individuals with HCC in our cohort. Tagatose is a monosaccharide and can be used as an alternative food sweetener. Only one other study identified a significant association with tagatose in the plasma of individuals with HCC, but they found tagatose to be elevated in the plasma of individuals with cirrhosis and lower in that of those with HCC. However, tagatose plasma levels have been shown to correlate with weight loss, and there is evidence that suggests that tagatose consumption may reduce the symptoms of metabolic syndrome [25]. Therefore, the relationship between tagatose, the development and progression of metabolic syndrome, cirrhosis, and HCC warrants further investigation.

### 4.3. Comparison of Salivary and Plasma Metabolite Profiles

Although there was no overlap in the metabolites in saliva and plasma that were significantly associated with HCC, we expanded this analysis to characterize the correlation between the full metabolic profiles of the two biospecimens. Irrespective of disease status, malonic acid was the only metabolite that was significantly correlated between the paired blood and saliva samples (*r* = 0.71). Malonic acid was also shown to be associated with HCC in the salivary samples but failed to reach statistical significance in the plasma. 

Coordinated shifts in metabolic abundances of peripheral fluids can be indicative of functional pathways and network dysregulation in malignant liver cells. We therefore investigated the disruption of pathways and networks in saliva and plasma.

### 4.4. Energy Pathways

The Warburg effect has been extensively studied and implicated as the major culprit for supplying energy to cancer cells [26]. This pathway was enriched for metabolites found in the saliva of individuals with HCC in our study. The ‘citric acid cycle’, also known as the TCA cycle, was also enriched for altered metabolites found in the plasma and saliva of individuals with HCC. Aberrant energy metabolism to sustain proliferation is a hallmark of cancer, and recent studies have implicated dysregulation of the TCA cycle in this process [27]. Another energy-generating pathway that was enriched in saliva of patients with HCC was the ‘mitochondrial electron transport chain’ (ETC), which generates energy through reduction of electrons, creation of a proton gradient across the mitochondrial membrane, and finally, oxidative phosphorylation of ADP to generate ATP. ETC is also an important source of reactive oxygen species (ROS), which are known to be an important hallmark of tumorigenesis [28]. ETC is an extremely efficient pathway; nevertheless, an estimated 0.2–2% of electrons passing through the chain escape and create ROS [28]. Tumors with mutations affecting the ETC are known to have strong tendencies to produce ROS [28]. Ubiquinol–cytochrome *c* reductase hinge (UQCRH), a subunit in complex III of the ETC, is overexpressed in HCC and is associated with ROS overproduction and poor prognosis [28,29].

### 4.5. Amino Acid Metabolism Pathways

‘Aspartate metabolism’, ‘glutamate metabolism’, ‘alanine metabolism’, ‘arginine and proline metabolism’, ‘histidine metabolism’, and ‘valine, leucine and isoleucine degradation’, were enriched for HCC-associated metabolites in saliva. Amino acids have been extensively studied for their role in cancer development and progression [30]. Glutathione (GSH) is a tripeptide synthesized from glutamate, cysteine, and glycine and is metabolized to produce the same. Alteration of GSH reactions, inhibiting removal and detoxification of carcinogens, can greatly affect cell survival, including providing a growth advantage to cancer cells [31]. Targeting GSH homeostasis holds great therapeutic potential in various cancer types [32,33] and detectable dysregulation of the glutathione metabolism pathway should be explored in the future to determine whether it can serve as a biomarker of therapeutic response to emerging GSH targeting interventions. Disruption of the ‘Alanine, aspartate and glutamate metabolism’ pathways may be indicative of hepatic injury. Alanine and aspartate metabolism is facilitated by alanine and aspartate aminotransferase (ALT, AST) enzymes, and high plasma ALT levels have been implicated in hepatic injury, steatosis, and metabolic syndrome without overt liver injury [34]. Out of all the metabolites associated with HCC, five metabolites, including succinic acid, citric acid, N-acetylaspartic acid, fumaric acid, and alpha-ketoglutarate, were enriched in ‘aspartate and alanine metabolism.’ Interestingly, two of the five metabolites, fumaric acid and alpha ketoglutarate, have been reported to aid tumor suppression in HCC in separate studies and thus have therapeutic potential [35,36,37], while succinic acid has been associated with resistance to Sorafenib [16]. Arginine metabolism has also been implicated in carcinogenesis as well as immune surveillance, a process involved in detecting and destroying cancerous cells [38,39]. Histidine is an amino acid that has not be extensively studied in association with malignancies. However, some studies suggest the utility of exogenous histidine in counteracting resistance to sorafenib in HCC [40]. In one study, exogenous histidine administration resulted in a decrease in the expression of tumor markers associated with glycolysis (GLUT1 and HK2), inflammation (STAT3), angiogenesis (VEGFB and VEGFC), and stem cells (CD133) [40]. High expression of these markers is associated with sorafenib resistance in HCC [40]. LAT1 (carrier protein responsible for histidine influx) was found to be downregulated in regions of high expression of the aforementioned markers [40]. Thus, dysregulation in the metabolism of histidine may play a crucial role in tumor activity. Branched-chain amino acids (BCAAs, i.e., valine, leucine, and isoleucine) provide nitrogen to synthesize macromolecules such as proteins and nucleotides and play a crucial role in cancer cell growth [41]. BCAA degradation generates various metabolites (e.g., glutamate) which feed other metabolic pathways implicated in carcinogenesis, including that of HCC [41,42]. In HCC, the dysregulation of BCAA catabolism causes accumulation of these amino acids in the tumor [42].

### 4.6. Lipid Metabolism Pathways

‘Fatty Acid Biosynthesis’ and ‘Long Chain Fatty Acids’ pathways were enriched for salivary metabolites associated with HCC when compared to cirrhosis + healthy. In addition to generating heat at the cost of ATP to maintain body temperature, these pathways produce a number of signaling molecules that regulate important biologic processes [43]. This centrality of the role of lipids at the structural and signaling levels means that any dysfunction of their metabolism results in a variety of pathophysiological conditions including metabolic syndromes (diabetes, obesity) [44], NASH, and cancer [45,46]. In contrast, calorie intake restriction leading to significant reduction in the storage of lipids in adipose tissue in addition to lipolysis, which is known to stop the age-related onset of metabolic diseases and slow aging [47,48,49].

### 4.7. Urea and Ammonia Metabolism

‘Ammonia recycling’ and ‘urea cycle’ were enriched in HCC with salivary metabolites. ‘Alanine, aspartate, and glutamate metabolism’, a key pathway for ammonia transport and recycling in the liver, was also enriched in those with HCC according to metabolites in their saliva. Urea is an end product of protein metabolism and functions to neutralize the ammonia produced by amino acid catabolism; it has been studied as a potential serum biomarker for HCC [50].

### 4.8. Limitations and Future Directions

This pilot study was limited by sample size. Although the sample sizes were small, the observed effect sizes were large enough to result in statistically significant findings. However, future studies that include more individuals in each disease group, and include additional disease groups such as NAFLD and NASH, will be needed. We reported all findings with an FDR *p* < 0.1 to provide a clear estimation for the probability of Type I errors. In this study, we tested for linear relationships between salivary and plasma metabolites; in the future, a larger cohort will allow for the assessment of potential non-linear relationships. We will validate our findings using targeted mass spectrometry, in which a set of standards are used to quantify each metabolite, providing the absolute abundance of each metabolite in either saliva or plasma. Several of the metabolites identified in this study are part of large pathways, such as the ‘Citric Acid Cycle’, and these individual metabolites may not be specific enough to HCC to serve as biomarkers. However, with additional validation and quantification, these individual metabolites may serve constituent biomarkers in a larger part of a larger, multi-modal biomarker signature.

## 5. Conclusions

In conclusion, this study aimed to identify metabolites in saliva and plasma that can serve as biomarkers for hepatocellular carcinoma. Our analysis revealed six metabolites in saliva and ten metabolites in plasma that were associated with HCC. Interestingly, malonic acid was the only metabolite that showed correlation between paired saliva and plasma samples and was also found to be associated with HCC in saliva. Pathway analysis revealed deregulation of the ‘Citric Acid Cycle’ in both blood and saliva. The lack of correlation between the metabolites in these two biospecimens suggest that they may serve as independent sources of information for detecting HCC, yet importantly, these non-overlapping metabolites converge on a common molecular pathway that is deregulated in states of carcinogenesis.

Our future studies will quantify these metabolites in saliva to better characterize the thresholds of metabolic abundance that can discriminate HCC from healthy or cirrhotic livers as we move towards a combined predictive model. This exploration of salivary and plasma metabolic profiles illuminates the unique contribution of salivary metabolites as potential biomarkers of HCC.

## Figures and Tables

**Table 1:** Summary statistics for study cohort.

Characteristic	Healthy ^1^	Cirrhosis	HCC
Total (*N*)	14	12	16
Mean age (min-max)	35.5 (19–56)	53.6 (32–66)	68.4 (41–83)
Sex			
Male (%)	7 (50%)	4 (33%)	11(55%)
Female (%)	7 (50%)	8 (67%)	5 (45%)
Diabetes mellitus type 2	0 (0%)	7 (58%)	6 (37%)
Hypertension	1 (7%)	7 (58%)	13 (81%)
Coronary artery disease	0 (0%)	2 (16%)	8 (50%)
Hyperlipidemia	2 (14%)	5 (41%)	4 (25%)
Psychiatric disorder	2 (14%)	3 (25%)	2 (12%)
COPD/asthma/OSA	1 (7%)	2 (16%)	5 (45%)
Other cancer history	0 (0%)	3 (25%)	2 (12%)
Thyroid	1 (7%)	3 (25%)	1 (6%)
Other PMH	1 (7%)	9 (75%)	8 (50%)
Ascites	0 (0%)	6 (50%)	1 (6%)
Encephalopathy	0 (0%)	2 (16%)	0 (0%)
Mean hemoglobin (g/dL) (SEM)	14.4 (0.27)	11.2 (0.79)	13.8 (0.46)
Mean platelets (k/uL) (SEM)	282.9 (14.6)	120.3 (18.9)	177.4 (17.1)
Mean AST (U/L) (SEM)	21 (1.6)	52 (8.3)	40.1 (4.7)
Mean ALT (U/L) (SEM)	22.5 (3.6)	45.6 (10.5)	37.3 (5.5)
Mean ALP (U/L) (SEM)	59.2 (4.2)	203 (44)	144.8 (51.1)
Mean bilirubin, Total (mg/dL) (SEM)	0.4 (0.06)	1.3 (0.15)	0.6 (0.12)
Mean albumin (g/dL) (SEM)	4.75 (0.06)	3.8 (0.12)	4.2 (0.07)
Mean PT-INR (SEM)	1 (0.01)	1.1 (0.06)	1 (0.01)
Mean glucose (mg/dL) (SEM)	87.2 (3.6)	142.9 (21)	129.5(11.6)
Mean creatinine (mg/dL) (SEM)	0.8 (0.03)	0.9 (0.07)	1(0.11)

^1^ Patient samples obtained from those being seen in the hernia clinic at Cleveland Clinic with no known liver disease.

## Data Availability

The data presented in this study are available in Supplementary Tables S5–S7.

## Supplementary Materials

The following supporting information can be downloaded at: https://www.mdpi.com/article/10.3390/cancers15184527/s1, Table S1: Metabolites removed due to <10% high-quality spectra counts in plasma or saliva. Table S2: Association of salivary metabolites with HCC. Table S3: Association of plasma metabolites with HCC, Table S4: Correlation of salivary and plasma metabolites, Table S5: Patient IDs and diagnoses, Table S6: Salivary metabolic abundances for named metabolites, Table S7: Plasma metabolic abundances for named metabolites. Figure S1: Distributions of metabolic abundances of plasma metabolites. Figure S2: Distributions of metabolic abundances of salivary metabolites. Methods S1.1: Mass spectrometry parameters. Methods S1.2: Detection of high-quality spectra. Methods S1.3: Hypergeometric Test.
