# Supplementary material for: Characterization of Salivary and Plasma Metabolites as Biomarkers for HCC: A Pilot Study
Source: Cancers (Basel). 2023 Sep 12;15(18):4527. doi: 10.3390/cancers15184527 (PMC10527521; doi:10.3390/cancers15184527)
Supplement: Supplementary file 1 [file cancers-15-04527-s001.zip › Supplementary_Materials.pdf]

Supplementary Figures

A)

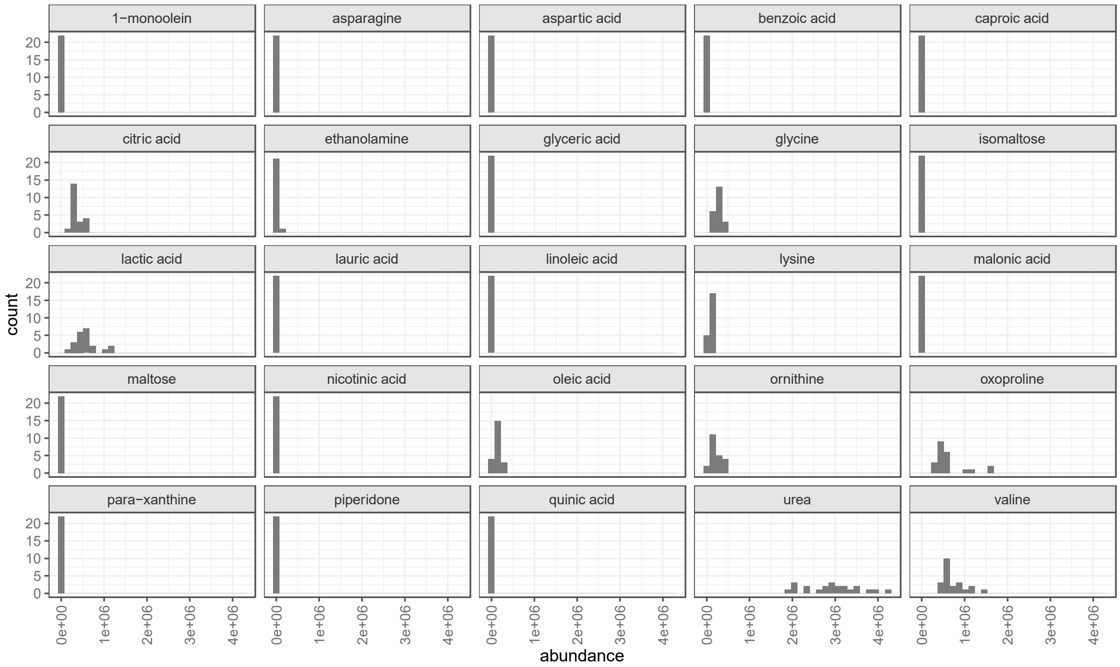

B)

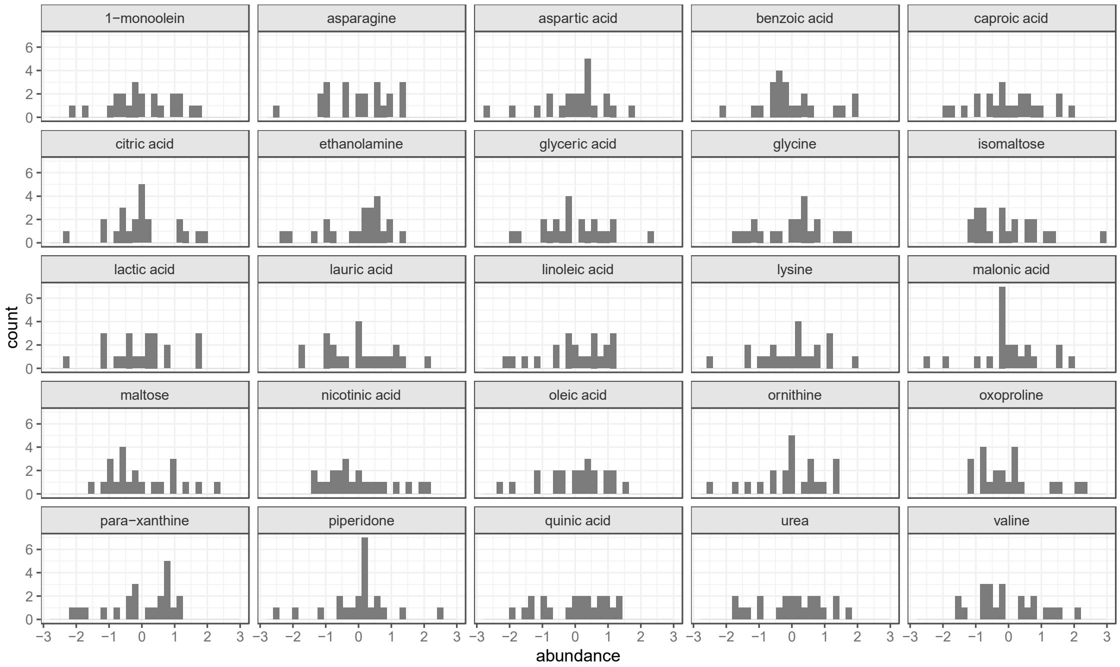

Figure S1: Distributions of metabolic abundance of plasma metabolites. A) Untransformed metabolite abundance of 25 plasma metabolites. **B)** Log_{10} transformation of 25 plasma metabolites.

A)

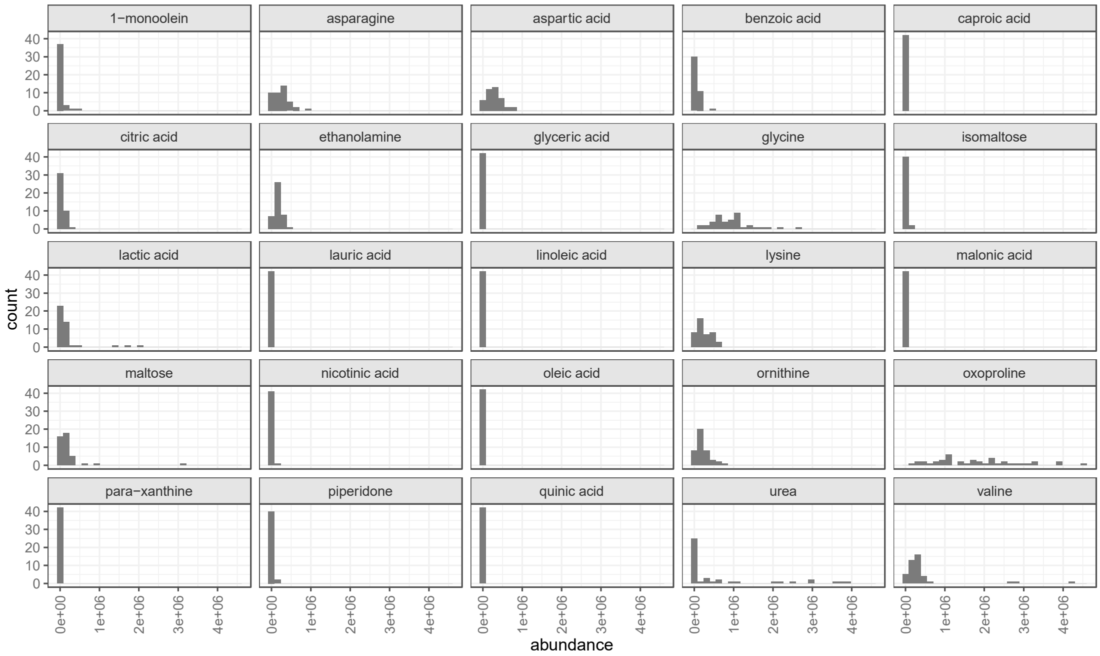

B)

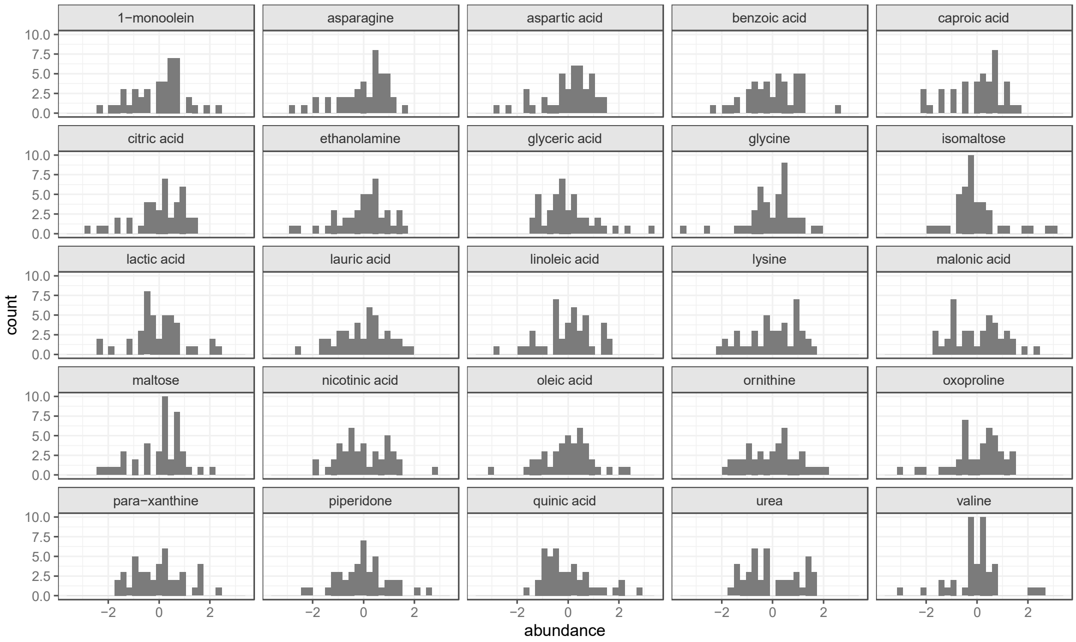

Figure S2: Distributions of metabolic abundance of salivary metabolites. A) Untransformed metabolite abundance of 25 plasma metabolites. **B)** Log_{10} transformation of 25 plasma metabolites.

Supplementary Methods

S1.1 Mass Spectrometry Parameters:

Mass spectrometry parameters are used as follows: a Leco Pegasus IV mass spectrometer is used with unit mass resolution at 17 spectra s-1 from 80-500 Da at -70 eV ionization energy and 1800 V detector voltage with a 230°C transfer line and a 250°C ion source.

S1.2 Detection of High Quality Spectra:

ChromaTOF version 2.32 was used for data preprocessing. It performed 3 s peak width and baseline subtraction followed by automatic mass spectral deconvolution and peak detection at signal/noise levels of 5:1. The apex masses were used in the BinBase algorithm. The BinBase algorithm (rtx5) filtered peaks based on the validity of the chromatogram (<10 peaks with intensity of >107 counts s-1), unbiased retention index marker detection (MS similarity >800), and retention index calculation by 5th order polynomial regression.

The Spectra were then cut to 5% base peak abundance and matched to the BinBase database using the retention index window (+-2s retention time), validation of unique ions and apex masses (>3% base peak abundance), peak purity, signal/noise ratios, and final isomer filter. The quantification for each metabolite was reported as peak height of the unique ion. Metabolites were reported if they had at least 10% of samples in either saliva or plasma that met these thresholds. Low confidence peaks were reported for samples in which high quality peaks were identified for at least 10% of samples within either saliva or plasma.

S1.3 Hypergeometric Test:

In short, M is the total number of metabolites, m is the number of nominally significant metabolites. S is the number of metabolites in the literature-annotated metabolite set. X is the number of nominally significant metabolites that that overlap with the metabolite set. The hypergeometric distribution samples without replacement and is used because the trials are not independent.

$$P(X \geq x) = 1 - P(X \leq x-1) = 1 - \sum_{i=0}^{x-1} \frac{\binom{S}{i}\binom{M-S}{m-i}}{\binom{M}{m}}$$
